# Association of *MTHFR* gene C677T mutation with diabetic peripheral neuropathy and diabetic retinopathy

**Published:** 2013-07-25

**Authors:** Serbulent Yigit, Nevin Karakus, Ahmet Inanir

**Affiliations:** 1 Gaziosmanpasa University, Faculty of Medicine, Department of Medical Biology, Tokat, Turkey; 2 Gaziosmanpasa University, Faculty of Medicine, Department of Physical Therapy and Rehabilitation, Tokat, Turkey

## Abstract

**Purpose:**

Diabetic peripheral neuropathy (DPN) is one of the most common diabetic chronic complications. Methylenetetrahydrofolate reductase (*MTHFR*) gene variants have been associated with vasculopathy that has been linked to diabetic neuropathy. The aim of the present study was to investigate the possible association between *MTHFR* gene C677T mutation and DPN and evaluate if there is an association with clinical features in a relatively large cohort of Turkish patients.

**Methods:**

The study included 230 patients affected by DPN and 282 healthy controls. Genomic DNA was isolated and genotyped using the polymerase chain reaction–based restriction fragment length polymorphism assay for the *MTHFR* gene C677T mutation.

**Results:**

The genotype and allele frequencies of the C677T mutation showed statistically significant differences between the patients with DPN and the controls (p=0.003 and p=0.002, respectively). After the patients with DPN were stratified according to clinical and demographic characteristics, a significant association was observed between the C677T mutation and history of retinopathy (p=0.039).

**Conclusions:**

A high association between the *MTHFR* gene C677T mutation and DPN was observed in the present study. In addition, history of retinopathy was associated with the *MTHFR* C677T mutation in patients with DPN.

## Introduction

Diabetes is a common disorder with various systemic complications including diabetic peripheral neuropathy (DPN), which affects most diabetic patients and causes sensory, motor, and/or autonomic dysfunctions [1,2]. Diabetic retinopathy is the most common complication of diabetes mellitus [3] and is rapidly emerging as a global health issue that may threaten patients' visual acuity and visual functioning. However, the underlying pathogenesis of DPN and diabetic retinopathy has not been well defined. It is widely accepted that vascular complication is the most common cause of diabetes death and disability and has been linked to diabetic neuropathy [4]. A risk factor for vasculopathy in diabetes is elevated homocysteine levels [5]. It has been reported that hyperhomocysteinemia in diabetic patients was associated with the prevalence of diabetic nephropathy and diabetic retinopathy [6,7]. Hyperhomocysteinemia was also independently related to the prevalence of diabetic neuropathy in diabetic patients [8-10].

Methylenetetrahydrofolate reductase (MTHFR) is the enzyme that catalyzes the transformation of homocysteine to methionine via the remethylation pathway (gene located in 1p36) [11]. Hyperhomocysteinemia is the consequence of decreased activity of MTHFR [12]. Mutations of the *MTHFR* gene lead to decreased enzymatic activity. The most common *MTHFR* variant is a point mutation (C→T substitution at nucleotide 677) resulting in an enzyme with 50% less activity [13]. The C677T mutation of the *MTHFR* gene, causing an amino acid change from alanine to valine, is associated with reduced activity and increased thermolability of the enzyme [14]. This mutation is the most common genetic cause of elevated homocysteine levels [15,16].

Although several studies showed that hyperhomocysteinemia is associated with diabetic neuropathy [8-10], no study has focused on *MTHFR* gene mutations in association with DPN. Thus, we decided to investigate the *MTHFR* gene C677T mutation in patients with DPN and evaluate if there is an association with clinical features in a relatively large cohort of Turkish patients with DPN.

## Methods

### Subjects

230 unrelated patients with DPN who were registered at the outpatient clinics of the Physical Therapy and Rehabilitation Department of Gaziosmanpasa University Tokat, Turkey, were included in the study (61 men, 169 women; mean age, 57 years old; age range, 17-83 years old). The patients all fulfilled the American Diabetes Association (ADA) criteria for diabetes (types 1 and 2) [17]. We used the standard Neuropathy Symptom Score (NSS) and Neuropathy Disability Score (NDS) criteria to diagnose diabetic neuropathy [18,19]. The patients underwent an ophthalmological examination, including visual acuity, slit-lamp examination, and funduscopy for the absence or presence of retinopathy. A total of 282 unrelated healthy subjects (mean age 55.550±8.149 years; 65 men, 217 women) were recruited consecutively. All participants, patients and healthy controls, were of Turkish origin, from the inner Central Black Sea region of Turkey. The healthy controls were matched for age, gender, and geographic area with patients with DPN and were free from another systemic disease. The protocol of this study was approved by the Institutional Ethics Committee, and all participants gave written informed consent before enrolling in the study.

### Genotyping

Genomic DNA was extracted from whole venous blood samples using a commercial DNA isolation kit (Sigma-Aldrich, Taufkirchen, Germany). The *MTHFR* C677T (rs1801133) mutation was analyzed with PCR–based restriction fragment length polymorphism (RFLP) assay as described previously [12]. The amplification conditions consisted of an initial melting step of 5 min at 94 °C; followed by 35 cycles of 30 s at 94 °C, 30 s at 61 °C, and 30 s at 72 °C; and a final elongation step of 5 min at 72 °C. The sequences of PCR primers were 5′-TGA AGG AGA AGG TGT CTG CGG GA-3′ and 5′-AGG ACG GTG CGG TGA GAG TG-3′. PCR was performed in a total volume of 25 µl reaction containing 100 ng of genomic DNA, 2.5 µl of 10X PCR buffer, 200 µM deoxynucleotide triphosphates, 10 pM each primer, and one unit of Taq DNA polymerase. After amplification, the 198 bp PCR product was digested with HinfI in a 15 µl reaction solution containing 10 µl of PCR product, 1.5 µl of 10X buffer, and two units of HinfI at 37 °C overnight. The digestion products were separated on 3% agarose gels, and fragments stained with the ethidium bromide were photographed on an ultraviolet transilluminator. Wild-type (CC) individuals were identified by only a 198 bp fragment, heterozygotes (CT) by the 175/23 bp and 198 bp, and homozygote variants (TT) by the 175/23 bp.

### Statistical analysis

Statistical analysis was performed using the Statistical Package for the Social Sciences (SPSS version 13.0, SPSS Inc., Chicago, IL) and the OpenEpi Info software package version 2.3.1. Results are given as mean±standard deviation (SD). The chi-square (χ^2^) test was used to evaluate the Hardy–Weinberg equilibrium for the distribution of the genotypes of the patients and the controls. The relationships between the C677T mutation and the clinical and demographics features of the patients were analyzed by using the χ^2^ test or analysis of variance (ANOVA) statistics. The χ^2^ test and Fisher’s exact test were used to compare categorical variables appropriately, and odds ratios and 95% confidence intervals were used to assess the risk factors. All p values were two-tailed, and p values less than 0.05 were considered statistically significant.

## Results

The baseline clinical and demographic features of the study patients with DPN are shown in Table 1. Gender, age, disease duration, height, weight, body mass index (BMI), hemoglobin, serum creatinine, glycated hemoglobin (HbA1C), triglyceride, total cholesterol, high-density lipoprotein (HDL) cholesterol, low-density lipoprotein (LDL) cholesterol, systolic blood pressure, diastolic blood pressure, type of diabetes mellitus, history of retinopathy, history of hypertension, medications, and smoking of patients were analyzed. Age and gender did not differ between the patient and control groups (p=0.055 and 0.409, respectively). Baseline characteristics of the patients according to the *MTHFR* genotypes were similar, except history of retinopathy. The frequency of the TT genotype was significantly higher in patients with a positive history of retinopathy than in the patients with a negative history of retinopathy (p=0.039; Table 1).

**Table 1:** Baseline clinical and demographical features of the 230 study patients with DPN.

Characteristic	Total n=230	CC n=123	CT n=85	TT n=22	P value
Gender, no. male/female (%)	61/169 (26.5/73.5)	32/91 (26.0/74.0)	24/61 (28.2/71.8)	5/17 (22.7/77.3)	0.858
Age (years)	57.15±10.580	57.46±11.440	56.21±9.912	59.09±7.708	0.472
Disease duration (years)	7.73±6.006	7.13±6.015	8.38±6.184	8.55±5.078	0.271
Height (cm)	162.84±6.753	162.61±6.807	163.82±6.973	160.00±4.410	0.073
Weight (kg)	78.53±9.208	78.42±9.684	78.16±8.941	80.74±7.504	0.540
BMI (kg/m2)	29.78±4.260	29.81±4.562	29.24±3.888	31.66±3.402	0.059
Hemoglobin (g/dl)	12.91±1.241	12.85±1.229	12.95±1.290	13.02±1.154	0.766
Serum creatinine (mg/dl)	0.80±0.232	0.79±0.239	0.82±0.238	0.75±0.145	0.332
HbA_1C_ (%)	6.97±1.668	7.04±1.891	7.04±1.442	6.29±0.839	0.128
Triglyceride (mg/dl)	166.44±80.057	160.29±54.746	179.25±110.787	151.36±47.530	0.159
Total cholesterol (mg/dl)	187.34±53.062	185.45±45.675	189.53±63.832	189.45±47.333	0.847
HDL cholesterol (mg/dl)	41.57±9.820	41.64±10.061	40.76±9.171	44.23±10.819	0.336
LDL cholesterol (mg/dl)	129.57±32.376	128.44±32.108	131.24±32.439	129.40±34.827	0.829
Systolic blood pressure (mmHg)	129.53±22.336	127.95±21.137	131.41±23.787	131.14±23.449	0.516
Diastolic blood pressure (mmHg)	81.10±14.550	80.15±14.718	82.47±14.467	81.14±14.136	0.529
Type of diabetes mellitus, no. Type 1/Type 2 (%)	5/225 (2.2/97.8)	3/120 (2.4/97.6)	2/83 (2.4/97.6)	0/22 (0/100)	0.762
History of retinopathy, no. Positive/Negative (%)	81/149 (35.2/64.8)	38/85 (30.9/69.1)	30/55 (35.3/64.7)	13/9 (59.1/40.9)	**0.039**
History of hypertension, no. (%)	129 (56.1)	65 (50.4)	50 (38.8)	14 (10.95)	0.524
Medications, no. Oral antidiabetic/Insulin (%)	177/38 (77.0/16.5)	91/23 (74.0/18.7)	65/15 (76.5/17.6)	21/0 (95.5/0)	0.242
Smoking, no. (%)	30 (13.1)	15 (50.0)	12 (40.0)	3 (10.0)	0.906

Mean plus standard deviation values are presented for all variables, except gender, type of diabetes mellitus, history of retinopathy, history of hypertension, medications and smoking. DPN: Diabetic peripheral neuropathy, BMI: Body mass index, HbA_1C:_ glycosylated hemoglobin, HDL: high density lipoprotein, LDL: low density lipoprotein. The results that are statistically significant are shown in boldface.

The frequency of the CC, CT, and TT genotypes of the C677T mutation in the patients was 53.5%, 37.0%, and 9.5%, respectively, and in the controls, the frequency was 63.8%, 33.0%, and 3.2%, respectively (Table 2). The C and T allele frequencies were 72.0% and 28.0%, respectively, in the patient group and 80.3% and 19.7%, respectively, in the control group (Table 2). The distributions of the genotype and allele frequencies of the *MTHFR* gene C677T mutation were statistically different between the patients with DPN and the control group (p=0.003 and p=0.002, odds ratio=1.59, 95% confidence interval =1.19–2.13; Table 2). High differences were also observed when the patients and the controls were compared according to CC versus CT+TT and CC+CT versus TT genotypes (p=0.018 and p=0.003, respectively; Table 2). The MTHFR gene C677T mutation was associated with DPN susceptibility in a Turkish population. The observed and expected frequencies of the mutation were in Hardy–Weinberg equilibrium in the patient and control groups.

**Table 2:** Genotype and allele frequencies of *MTHFR* gene C677T polymorphisms in patient and control groups.

***MTHFR***	**DPN patients**	**Healthy controls**	p	OR (CI 95%)
**C677T**	**n=230 (%)**	**n=282 (%)**		
Genotypes				
CC	123 (53.5)	180 (63.8)	0.003	
CT	85 (37.0)	93 (33.0)		
TT	22 (9.5)	9 (3.2)		
CC+CT: TT	208 (90.4): 22 (9.6)	273 (96.8): 9 (3.2)	0.003	3.20 (1.47–7.46)
CC: CT+TT	123 (53.5): 107 (46.5)	180 (63.8): 102 (36.2)	0.018	1.53 (1.07–2.19)
Alleles				
C	331 (72.0)	453 (80.3)	0.002	1.59 (1.19–2.13)
T	129 (28.0)	111 (19.7)		

MTHFR: methylene tetrahydrofolate reductase, DPN: Diabetic peripheral neuropathy. The results that are statistically significant are shown in boldface.

## Discussion

The major finding of the present study is the demonstration of an association between the *MTHFR* gene C677T mutation and DPN as well as history of retinopathy. In vitro studies showed that hyperhomocysteinemia affected nervous function by direct cytotoxic effects or by oxidative damage of endothelial cells, leading to occlusive arteriosclerosis in small vessels [20,21]. Therefore, macro- and microvascular damage associated with higher hyperhomocysteinemia plasma values could be associated with nerve damage and would explain our results.

In a previous study, the total plasma homocysteine concentrations and the frequency of hyperhomocysteinemia were significantly higher in the group of diabetic patients with neuropathy compared to non-neuropathic patients in a German population (p=0.04) [8]. In the same study, the vitamin B_12_ concentrations demonstrated a trend to decrease in the neuropathy group (p=0.06). Plasma total homocysteine concentrations were also independently associated with the occurrence of diabetic neuropathy in two recent Chinese studies [9,10]. In these Chinese studies, the plasma homocysteine levels were significantly higher in patients with diabetic neuropathy than in patients without diabetic neuropathy (p<0.001 and p=0.005, respectively). The authors hypothesized that hyperhomocysteinemia was an independent risk factor for the occurrence of diabetic neuropathy [9,10].

Diabetic retinopathy is a leading cause of blindness [22]. Hyperhomocysteinemia has been associated with diabetic retinopathy [23]. In vitro studies indicated that homocysteine increases the expression of the vascular endothelial growth factor in cell cultures via activation of its transcription [24,25]. The vascular endothelial growth factor is a proangiogenic factor known to play a key role in the development and progression of diabetic retinopathy [25,26]. The C677T mutation in the *MTHFR* gene has been related to homocysteine levels and to vascular diseases [12]. The relationship between this mutation and diabetic retinopathy has been evaluated in many studies. As a result of a recent meta-analysis that included eight case-control association studies, the 677TT genotype was associated with a moderately augmented risk for diabetic retinopathy [27]. In the current study, in concordance with the previous results, the frequency of the TT genotype was significantly higher in patients with a positive history of retinopathy than in patients with a negative history of retinopathy (p=0.039).

In conclusion, for the first time, our findings demonstrate that the *MTHFR* gene C677T mutation is related to DPN. The C677T mutation is also associated with a history of retinopathy in these patients. Our results showed that patients with the C677T mutation have a predisposition to DPN. Nevertheless, the significance of the *MTHFR* gene C677T mutation in DPN requires further studies.
